# Mitochondrial Metabolism in Major Depressive Disorder: From Early Diagnosis to Emerging Treatment Options

**DOI:** 10.3390/jcm13061727

**Published:** 2024-03-17

**Authors:** Ane Larrea, Laura Sánchez-Sánchez, Eguzkiñe Diez-Martin, Ane Elexpe, María Torrecilla, Egoitz Astigarraga, Gabriel Barreda-Gómez

**Affiliations:** 1 Research and Development Division, IMG Pharma Biotech, 48170 Zamudio, Spain; anelarrea96@gmail.com (A.L.); laura.sanchez@imgpharma.com (L.S.-S.); eguz@imgpharma.com (E.D.-M.); ane@imgpharma.com (A.E.);; 2 Department of Pharmacology, Faculty of Medicine and Nursing, University of the Basque Country UPV/EHU, 48940 Leioa, Spain; maria.torrecilla@ehu.eus; 3 Department of Immunology, Microbiology and Parasitology, Faculty of Science and Technology, University of the Basque Country UPV/EHU, 48940 Leioa, Spain

**Keywords:** Major Depressive Disorder, mitochondrial dysfunction, ketamine/esketamine, psychedelic, inflammation, transcranial stimulation

## Abstract

Major Depressive Disorder (MDD) is one of the most disabling diseases in the world. MDD is traditionally diagnosed based on a patient’s symptoms, which can lead to misdiagnosis. Although the pathogenic mechanisms of MDD are unknown, several studies have identified mitochondrial dysfunction as a central factor in the onset and progression of MDD. In the context of MDD, alterations in mitochondrial metabolism can lead to imbalances in energy production and oxidative stress, contributing to the disorder´s underlying pathophysiological mechanisms. Consequently, the identification of mitochondrial dysfunction as a key biomarker for early and accurate diagnosis of MDD represents a significant challenge. Faced with the limits of traditional treatments with antidepressants, new pharmacological therapeutic targets are being investigated such as ketamine/esketamine, psychedelics, or anti-inflammatories. All of these drugs show potential antidepressant effects due to their speed of action and ability to modulate neuroplasticity and/or motor processing. In parallel, non-pharmacological therapeutic targets are studied, like Transcranial Magnetic Stimulation (TMS) and Deep Brain Stimulation (DBS), recognized for their ability to modulate neuronal activity and offer treatment alternatives. As cellular activity is directly related to mitochondrial respiration, the aim of this review is examining the link between mitochondrial dysfunction and MDD, assessing how mitochondrial biomarkers could provide a more objective and precise diagnostic tool, and exploring other treatments in addition to traditional antidepressants, with a specific focus on emerging therapeutic targets. Finally, a detailed analysis of the strengths, weaknesses, opportunities, and threats of these approaches was carried out, highlighting the key challenges that must be addressed.

## 1. Introduction

Depressive disorders are one of the most disabling mental health problems. They are characterized by symptoms such as abnormal depressive mood, irritability, fatigue, and the diminished ability to concentrate, among others [1]. This pathology is diagnosed by a clinical interview using the criteria from the Diagnostic and Statistical Manual of Mental Disorders (DSM-5). To evaluate the severity of this illness along with the response to antidepressant treatment, there are various tools, such as the Beck Depression Inventory II (BDI-II), Hamilton Rating Scale for Depression (HRSD), or Montgomery Asberg Depression Rating Scale (MADRS) [2]. Those rating scales can detect the presence of depressive symptoms and evaluate their severity. Inside the group of depressive disorders, we can distinguish between Persistent Depressive Disorders, Other Specified Depressive Disorders, or Major Depression Disorders (MDD), among others. Major depression symptoms include culpability, suicidal ideation, and loss of cognitive functions along with the general symptoms described above. Despite the prevalence of depression, the etiopathogenesis is still unclear, due to the difficulty of observing pathological changes in brain tissues, and the limitation of neuroimaging techniques [3]. Treatment for depression is established based on the severity of the illness and is often stepped, although unfortunately some patients do not respond satisfactorily to treatment. In milder cases of the illness, treatment focuses on support, psychoeducation, and psychological interventions such as cognitive-behavioral therapy, behavioral activation, or problem-solving therapy, which may sometimes be accompanied by antidepressant treatment. In patients with moderate depression, antidepressant treatments are usually required in addition to psychological interventions. When there is an insufficient response to two or more trials with different classes of antidepressants using optimal doses, the patient is considered to have treatment-resistant depression (TRD) [4]. In such cases, additional strategies are needed to address the problem and alleviate the patient’s depressive symptoms. One of the therapeutic options available is to increase the dose of the antidepressant used, switch to another antidepressant, or combine them with other medications [5].

### 1.1. Monoamine Deficiency 

The main mechanism of action of most current antidepressants is based on increasing the levels of certain monoamines during synapses, according to the monoaminergic deficit theory of depression [6]. This hypothesis suggests that depression is related to imbalances in the activity of neurotransmitters in specific regions of the brain, leading to decreased concentrations of serotonin and noradrenaline [6]. More specifically, levels of 5-hydroxyindoleacetic acid, a serotonin metabolite, are reduced in the cerebrospinal fluid (CSF) of depressive patients. However, there are not only brain alterations; interestingly, serotonin uptake is also reduced in platelets. Along with this, the action of antidepressants that increase the levels of 5-hydroxytryptamine (serotonin) or dopamine seems to reduce depressive symptoms [7]. 

Various types of antidepressants address the monoaminergic deficit theory, including monoamine oxidase inhibitors (MAOIs), which increase overall monoamine levels; tricyclic antidepressants (TCAs), which target the monoaminergic system with mixed effects and side effects; selective serotonin reuptake inhibitors (SSRIs), such as fluoxetine, which specifically increase serotonin; and serotonin-norepinephrine reuptake inhibitors (SNRIs), which increase both serotonin and noradrenaline [8]. In particular, SSRIs are the first-line medications for the pharmacological treatment of MDD. Within this group are fluoxetine, citalopram, escitalopram, paroxetine, fluvoxamine, and/or sertraline. This class of drugs shows lower affinity than TCAs and MAOIs for other receptors, which often results in a higher safety profile with less adverse effects [9].

In this context, the 5-HTPPLR polymorphism has emerged as a significant area of interest in depression research and response to treatment with SSRIs. This polymorphism refers to a specific genetic variant that affects serotonin receptors in the brain. It has been suggested that variations in this polymorphism may influence individuals’ response to SSRIs, which could have important implications for personalized depression treatment [10]. Research on the 5-HTPPLR polymorphism and its relationship with response to SSRIs has yielded mixed results and is not yet fully understood. Some studies have suggested that certain genetic variants may be associated with greater efficacy of SSRIs in treating depression [11]. However, others indicate that there is no association between the polymorphism and MDD [12]. 

However, it is important to note that the monoaminergic deficit theory of depression and research on the 5-HTPPLR polymorphism are not without criticism and controversy. Some experts have questioned the validity and clinical relevance of this theory, arguing that the underlying mechanisms of depression may be much more complex and multifactorial than the monoaminergic theory suggests [13]. Additionally, other neurochemical and neurobiological systems may also play an important role in the pathophysiology of depression and response to treatment, such as the glutamatergic system, the endocannabinoid system [14], and the opioid peptide system [15].

### 1.2. Mitochondrial Hypothesis 

The human brain is one of most energy-demanding organs that needs a continuous energy supply to maintain its function, on account of being the energy synthesis indispensable for different neurophysiological processes such as neuronal communication, as demonstrated by the high concentration of mitochondria present in dendrites and synaptic terminals [16].

**Figure 1:**
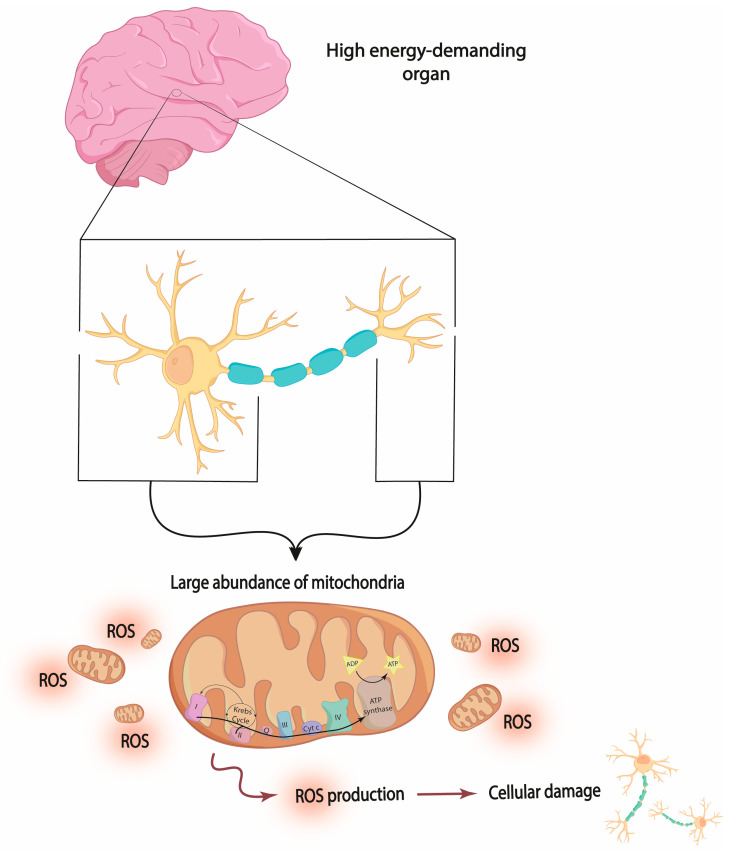
Representation depicting the mitochondrial hypothesis. The high density of mitochondria in the dendrites and synaptic terminals of neurons can generate a significant amount of ROS in pathological conditions that can alter the neuron functions. Inadequate antioxidant and anti-inflammatory activities may result in ROS accumulation, potentially leading to cellular damage. ROS: reactive oxygen species.

The mitochondrion is the organelle responsible for aerobic respiration used in the synthesis of ATP through the process of oxidative phosphorylation (OXPHOS) [17]. The inner membrane contains the mitochondrial electron transport chain (mETC) complexes that together with ATP synthase produce the ATP necessary to maintain cellular homeostasis. However, the high energy demand required for physiological neuronal activity can boost the formation of reactive species. When the reactive species exceed the antioxidant capacity, oxidative stress is produced, triggering cellular damage [18]. Reactive species can be produced from nitrogen or oxygen molecules. Reactive oxygen species (ROS) entails pathological processes of oxidative stress [6]. ROS arise from incomplete oxygen reduction [19] and can be produced in a variety of pathways, with mETC as the main source [9] (Figure 1). Thus, alterations in mitochondrial metabolism can cause imbalances in energy production and oxidative stress, contributing to the pathophysiological mechanisms underlying different neurological disorders such as MDD [20].

### 1.3. Mitochondrial Stress and Oxidative Damage in Depressive Disorders

The cellular homeostasis is highly dependent on the energy production, and thus on mitochondrial respiration. Mitochondrial dysfunctions are present in different neurological disorders such as MDD, not only in the central nervous system but also in peripheral tissues. In this sense, the expression of mitochondrial complex I was found to be reduced in neurons from depressed patients [21]. A reduction in the mitochondrial activity and oxygen consumption was also observed in blood cells of major depressive and bipolar disorder patients [22]. Moreover, a lower respiration capacity was detected in peripheral blood mononuclear cells (PBMCs) from depressed patients, which correlates in a negative manner with illness severity [23]. 

In addition to their main effects, antidepressants can alter energy metabolism leading to mitochondrial toxicity, which may increase the progression of existing diseases. Various antidepressants and mood stabilizers can inhibit different components of the mETC [24,25], increasing ROS production and reducing antioxidant capacity [26]. This energetic deficit and oxidative stress may result in cellular damage, such as that found in the cerebral areas of the pre-frontal cortex and hippocampus [27]. 

In depressive patients, mitochondrial complex I subunits are less expressed in neurons and complexes II and IV are less active in platelets [1]. In addition to those changes, a volume reduction in the frontal cortex and hippocampus has been detected in depressive patients, which can be related to oxidative stress-mediated apoptosis [28]. Moreover, mouse depressive models exposed to chronic mild stress display mitochondrial dysfunction and oxidative damage in cortex, hippocampal, and hypothalamic neurons [29]. Oxidative stress conditions can produce changes in cell membranes, proteins, genes, and lipids, altering the signal transduction and structural plasticity, among others [30]. Indeed, lipid peroxidation induced by ROS is a process that affects the lipid composition of membranes and the functionality of different transmembrane proteins including respiratory complexes and supercomplexes [31]. 

## 2. Depression Biomarkers

The diagnosis and identification of MDD patients’ remission are dependent on clinical evaluation and the lack of good biomarkers leads to under-diagnosis. Therefore, the identification of biomarkers that may be able to diagnose this illness or predict the future treatment response is crucial [32]. A good biomarker by definition is one that can be quantified from a sample that is easy to obtain, so most studies are focused on finding biomarkers in liquid biopsies such as CSF or blood [32]. In this regard, blood biomarkers have been classified into two different groups depending on their purpose: diagnosis or prognosis biomarkers. 

### 2.1. Diagnosis Biomarkers

The first problem when facing MDD diagnosis is the lack of a clear biomarker. Today, patients are not diagnosed unless they suffer sufficient symptoms to prompt them to consult a clinician, when a good blood biomarker could facilitate diagnosis in the early stages of the disease, even months before they meet the diagnostic criteria [33]. Some proteins have been reported in multiple studies as potential biomarkers for major depression. In this regard, a reduction in the concentration and expression of nocturnal melatonin [34], somatotropin [18], and brain-derived neurotrophic factor (BDNF) [35] was found in major depression patients. Moreover, differences on the methylation profile at the promoter of exon I in the BDNF gene were observed when comparing major depressive patients with healthy controls [33,36]. Specific biomarkers studied in PBMCs, such as inflammatory cytokines, seem to be increased in MDD patients [37]. These inflammatory processes may be directly linked to alterations in mitochondrial functionality and ROS formation in MDD. In this context, both ATP production in muscle [38] and mitochondrial respiration in platelets [39] have been reported to be decreased in depressed patients, becoming promising diagnostic biomarkers for this pathology [23]. 

### 2.2. Prognostic Biomarkers

As explained above, the immune system and MDD are closely related, allowing for the use of certain cytokines as a diagnostic biomarker such as IL-6, which is increased in patients with treatment-resistant major depression. In this sense, higher levels of this cytokine predicted a better response to ketamine therapy in TRD, whereas with metyrapone and SSRI the behavior was opposite [40]. Other prognostic biomarkers have been studied along with ketamine treatment, such as adiponectin, protein p11, or D-serine concentration which can predict the antidepressant response to ketamine [41,42]. Moreover, decreased concentrations in urine of 3-methosy-4-hydroxyphenylglycol, the major norepinephrine metabolite, correlate with the responsive capacity to imipramine treatment [43], and other tricyclic and tetracyclic antidepressants. Furthermore, serological superoxide dismutase activity was found to be reduced in patients with different antidepressant classes [44]. However, the effect of antidepressant medication on superoxide dismutase activity is unclear and further studies must be performed [45].

## 3. Treatment Options for Treatment-Resistant Depression

### 3.1. Pharmacological Treatment 

#### 3.1.1. Ketamine

Over the past few years, ketamine has emerged as a groundbreaking drug in current psychiatry, as it produces a rapid and long-lasting antidepressant effect in severely depressed patients [46]. It was first authorized in 1970 as a fast-acting anesthetic. It is composed of the enantiomers (R)-ketamine and (S)-ketamine and in addition to its anesthetic action, it also has analgesic and anti-inflammatory effects [47]. Ketamine is a derivative of phencyclidine, and norketamine ((R,S)-NK) and (2R,6R;2S,6S)-hydroxynorketamine (HNK) are its main active metabolites [47]. The first evidence of ketamine’s antidepressant effect dates back to the 1970s, but in 2000 it was revealed that subanesthetic doses administered intravenously via 40 min infusions produced an antidepressant effect compared to placebo within 24 h, which decreased after 7 days. Since then, numerous studies have demonstrated the efficacy of this drug in alleviating depressive symptoms [48,49,50]. While intravenous ketamine is not approved for the treatment of resistant depression, in this context, countries allow its individual use on compassionate grounds.

Although it should be noted that there is great variability (29–90%) in the number of patients responding to ketamine in the short term and concerns about long-term safety and tolerability require further investigations, in recent years the use of ketamine as an antidepressant has significantly increased, particularly in private clinics [51]. In this scenario, ketamine has been a remarkable discovery, not only due to the characteristics of its antidepressant effect but also because of the novelty of the proposed mechanism of action, as ketamine is not a monoaminergic antidepressant but rather an NMDA receptor antagonist that acts on various neuronal pathways including the opioid, monoaminergic, glutamatergic, and muscarinic systems, as well as substance P and sigma receptors [52]. NMDA receptors are heterotetramers that act as ion channels. Their activation requires the simultaneous binding of L-glutamate and glycine/D-serine to the GluN2 and GluN1 subunits, respectively, as well as the voltage-dependent displacement of Mg^2+^ from the ion channel pore, resulting in the entry of calcium into the neuron [53]. Several hypotheses have been described regarding the mechanism of action of ketamine and its metabolites, including direct action on NMDA receptors in glutamatergic neurotransmission, inhibition of NMDA receptors in GABAergic neurons, and energy metabolism [54]. The direct inhibition of extrasynaptic NMDA receptors causes the selective blockade of NMDA receptors containing extrasynaptic Glu2B, which are activated when there are low levels of environmental glutamate regulated by the glutamate transporter 1 (GLT-1) located in astrocytes. Ketamine’s inhibition of these receptors can activate mammalian targets of rapamycin (mTOR), which in turn induces protein synthesis. It has also been proposed that blocking the spontaneous activation of NMDA receptors by ketamine results in the inhibition of eukaryotic elongation factor 2 kinase (eEF2K) activity, thereby preventing the phosphorylation of its substrate, eukaryotic elongation factor 2 (eEF2). This ultimately leads to increased translation of BDNF, promoting protein synthesis and synaptogenesis. Importantly, while ketamine is a fast-acting antidepressant with long-lasting effects, its elimination half-life is short. In this regard, it has also been demonstrated that ketamine directly inhibits excessive NMDA receptor-dependent activity of lateral habenula (LHb) neurons associated with depressive symptoms [55]. Moreover, a recent study shows that the antidepressant effect is due to the use-dependent trapping of ketamine in NMDA receptors, making for significant progress in the understanding of the mechanism of action of new antidepressant agents [56].

**Figure 2:**
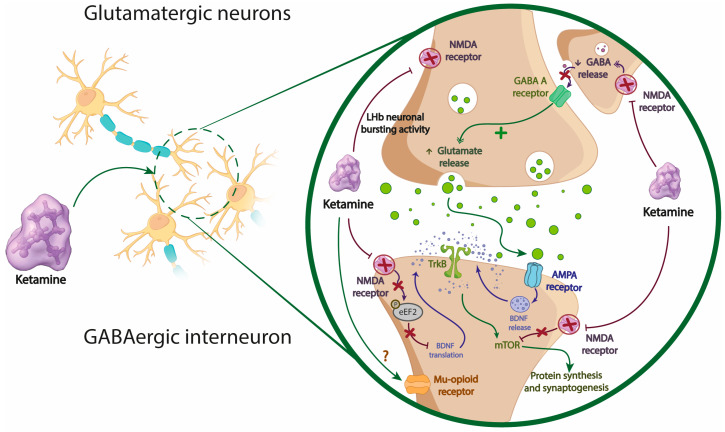
Representation of the mechanism of action of ketamine. Ketamine inhibits NMDA receptors, thereby facilitating the release of glutamate, which subsequently binds to AMPA receptors. Upon activation of AMPA receptors, there is an upregulation in BDNF release, subsequently activating TrkB receptors, ultimately culminating in protein synthesis and synaptogenesis through mTOR pathway activation. Other proposed mechanisms include indirect activation of µ receptor and activity-dependent blockade of NMDA receptors in LHb neurons. BDNF: brain-derived neurotrophic factor; LHb: Lateral Habenula; mTOR: mammalian target of rapamycin; NMDA: n-methyl-D-aspartate; TrkB: tropomyosin receptor kinase B; AMPA: AMPA (α-amino-3-hydroxy-5-methyl-4-isoxazolepropionic acid receptor.

The disinhibition hypothesis (Figure 2) proposes that ketamine selectively blocks NMDA receptors expressed in inhibitory GABAergic interneurons, leading to disinhibition of pyramidal neurons and increased glutamatergic firing [54]. The released glutamate binds to postsynaptic AMPA receptors, activating them, increasing BDNF release, which in turn activates tropomyosin receptor kinase B (TrkB), ultimately resulting in protein synthesis and synaptogenesis through mTOR activation. Lastly, the resulting HNK from ketamine metabolism suggests that ketamine exerts independent antidepressant actions via NMDA receptor inhibition through its metabolites, (2R,6R)-HNK and (2S,6S)-HNK [57]. Ketamine is metabolized into HNK after administration, and these metabolites enhance synaptic plasticity mediated by AMPA receptors. It should be noted that the proposed mechanisms of action may complement each other to exert ketamine’s antidepressant action by promoting synaptic plasticity and synaptogenesis [58]. In addition to glutamatergic effects, ketamine acts on the opioid system, which may also contribute to its antidepressant effects [59]. It has recently been demonstrated that activation of this system is necessary for the antidepressant effect of ketamine, where several studies show that opioid receptor antagonism reduces the antidepressant effect of ketamine. Recent studies suggest that although ketamine does not appear to act directly on µ opioid receptors, the functionality of these receptors is necessary for the drug’s antidepressant effects [59,60]. However, µ receptor activation does not mimic the cellular effects of ketamine.

Concomitant use of antidepressants appears to be safe and well-tolerated, although special caution is recommended with MAOIs, BZDs, and other anticonvulsant and sedative medications, as these may interfere with ketamine response. Additionally, a reduction in suicidal ideation has been observed persisting for up to 6 weeks in patients receiving intravenous racemic infusion [61,62]. However, it should be noted that the consequences of adverse effects are based solely on studies of a single administration of the drug, as there is insufficient data to analyze the safety of repetitive ketamine administrations or its long-term adverse effects. In this regard, the potential risk of long-term use, in addition to cardiovascular problems, would be the risk of developing dependence on repeated ketamine use [62,63].

##### Ketamine and Mitochondria

Recent studies indicate that ketamine not only acts at the synaptic level but also alters the main pathways of energy metabolism. The stages of glycolysis and the Krebs cycle consist of a series of biochemical reactions that generate molecules with high reducing power (NADH and FADH2) to produce high levels of energy in the mitochondria through OXPHOS [64]. It is important to mention that these two stages are not independent of each other, but the pathways of the mitochondrial matrix are linked to the pathways of oxidative phosphorylation. In this sense, ketamine alters the levels of certain metabolites in both glycolysis and the Krebs cycle [54]. Moreover, this drug leads to increased subunits of complex I and II, which in turn correlates significantly with behavioral studies after ketamine administration. Likewise, its action has been related to a rapid reduction in ROS and degradation of damaged proteins, favoring cellular homeostasis [65]. In addition, studies in animals have corroborated that the synaptogenesis observed in the prefrontal cortex circuit, a process that requires much energy, plays an important role in maintaining the antidepressant effect and not in its onset. Ketamine alters glutamatergic neurotransmission, which is in turn linked to energy metabolism, since the synthesis of glutamate begins in the Krebs cycle, the stage prior to OXPHOS. Simultaneously, this neurotransmission can be altered by the action of proinflammatory mediators regulated by the immune system. These mechanisms of action of ketamine are not mutually exclusive and can act complementarily in the exercise of the antidepressant actions of this drug [22,64]. It is important to note that ketamine also regulates the activity of the immune system and chronic inflammation, factors that appear to play an important role in the pathogenesis of depression. Both mitochondrial oxidative damages produced at the neuronal level and mitochondrial dysfunction observed in PBMCs can trigger uncontrolled inflammatory responses, producing proinflammatory mediators that ultimately affect neurotransmission and synaptic plasticity. In this sense, it has been observed that ketamine produces an anti-inflammatory effect by reducing the levels of certain proinflammatory cytokines and tumor necrosis factor (TNF-α) in patients with depressive disorders [66]. 

#### 3.1.2. Esketamine

With the aim of improving antidepressant efficacy and avoiding adverse effects, the enantiomers of ketamine, (S)-ketamine (esketamine) and (R)-ketamine, were also studied [67]. Consequently, the Food and Drug Administration (FDA) and EMA (European Medicines Agency) authorized the use of the nasal spray formulation of esketamine for the treatment of TRD, marketed under the brand name Spravato^®^ [68]. The FDA also approved intranasal esketamine for treating adults with major depression and suicidal ideation or behavior [69].

Esketamine is typically administered in combination with other antidepressants, such as SSRIs or SNRIs. This treatment strategy has been shown to be effective in patients with TRD [70]. In fact, clinical studies have revealed that adding esketamine to traditional antidepressant therapy can significantly increase the likelihood of achieving acute remission of depressive symptoms, representing a notable advance in the management of this disease [71].

The mechanism of action of esketamine involves the stimulation of the α-amino-3-hydroxy-5-methyl-4-isoxazolepropionic acid (AMPA-R) receptor, leading to increased neurotrophic signaling in brain regions involved in mood regulation and emotional behavior [72]. Additionally, it has been observed that esketamine can activate opioid receptors (mu, kappa, and delta), although its relationship with the antidepressant effect is not fully understood [67]. It seems that opioid direct activation appears to be independent of the antidepressant effect, as it has been shown that the rs1799971 (A118G) polymorphism, which alters opioid receptor agonist-mediated response, does not modulate esketamine’s effect [73]. However, it has been described that through this mechanism of action, endogenous opioid peptides that are involved in antidepressant functions may be released [74]. Furthermore, genetic studies have identified genes such as IRAK3 and NME7 that could influence esketamine’s efficacy, suggesting their involvement in processes related to immune response, stress regulation, and neuronal connectivity [75,76]. In addition to improving depressive symptoms, esketamine treatment has also shown improvements in other aspects of mental health, such as insomnia, and is not associated with weight gain [77], making it especially attractive for certain patients. However, the persistence of the antidepressant effect in the medium and long term is a topic of debate in the literature. Some studies have found that the improvement observed during the induction phase with esketamine is maintained during the maintenance phase [71], while others report that the benefits may not be as consistent over time [78,79]. These discrepancies may be due to differences in study design and the heterogeneity of the patient sample, highlighting the need for more research in this area.

##### Esketamine and Mitochondria

Esketamine also exerts its action through the modulation of mitochondrial function, a crucial aspect in cellular metabolism and ATP production. It is important to note that the proposed mechanisms of action do not act in isolation but are interconnected and may act synergistically to mediate the therapeutic effects of esketamine.

Esketamine has been shown to be capable of modifying central metabolic pathways, such as glycolysis and the Krebs cycle. This drug can influence glycolysis and the Krebs cycle in several ways. For example, studies have suggested that esketamine can modulate the enzymatic activity of key enzymes in these metabolic pathways [66]. The interaction of esketamine with enzymes such as phosphofructokinase in glycolysis and various dehydrogenases in the Krebs cycle can alter the speed and efficiency of these metabolic pathways, thus affecting ATP production in the mitochondria. Additionally, esketamine has been observed to regulate the gene expression of enzymes involved in glycolysis and the Krebs cycle. Changes in the expression of genes encoding key enzymes in these metabolic pathways could have a significant impact on the quantity and activity of these enzymes, which in turn could influence ATP production in the mitochondria. It may also interfere with the translocation of substrates and products across cell and mitochondrial membranes [22]. These transporters are crucial for the proper flow of metabolites through metabolic pathways, and any alteration in their function could affect the availability of substrates for ATP production in the mitochondria [80,81]. Additionally, it has been suggested that esketamine could modulate cellular signaling pathways that regulate metabolic activity, such as the insulin pathway or the insulin-like growth factor (IGF) pathway. These signaling pathways can influence gene expression and enzymatic activity in glycolysis and the Krebs cycle, which in turn can affect ATP production in the mitochondria [82].

On the other hand, an important aspect of esketamine is the modulation of respiratory complexes. Various studies have revealed that esketamine can influence the expression of subunits of these complexes, including complex I and II. These alterations could affect the efficiency of electron transport, which in turn could influence ATP production [65,66,83]. Additionally, esketamine has been shown to reduce ROS levels. Since mitochondria are a significant source of ROS, esketamine’s ability to reduce these levels suggests potential mitochondrial protection. This reduction in ROS could preserve mitochondrial function and prevent oxidative stress related to disease. Furthermore, esketamine has demonstrated anti-inflammatory properties and can reduce levels of proinflammatory cytokines. By modulating the inflammatory response, ketamine could indirectly protect mitochondrial function and prevent cellular damage associated with chronic inflammation [66].

#### 3.1.3. Psilocybin

The use of psychedelics has sparked growing interest in research as a potential treatment option for MDD, particularly psilocybin, which acts as an agonist of serotonergic 5-HT2A receptors and secondarily of 5-HT2B and 5-HT2C receptors [84]. The complex interactions of psilocybin with neurotransmitter release and intracellular signaling pathways suggest mechanisms of action that may impact synaptic plasticity and brain connectivity, offering innovative perspectives [85]. Research in this area is still in its early stages, although Australia has allowed the prescription of psilocybin as a treatment for TRD and post-traumatic stress disorder since July 2023 [86]. However, the pass patterns are not universal and can vary depending on study design, administered dosage, and individual characteristics. Furthermore, the interpretation of these changes in brain activity in relation to subjective experience and cognitive effects is still evolving. Therefore, more evidence is required before considering it an established therapy. 

The modulation of neuronal activity through the activation of 5-HT2A receptors induced by psilocybin triggers a series of events affecting neuronal communication and neurotransmitter release [87]. Psilocybin induces an increase in glutamate release due to the activation of 5-HT2A receptors, which could enhance synaptic transmission and contribute to the formation of new synaptic connections, thereby influencing brain activity [88]. Additionally, the signaling cascade initiated by the activation of 5-HT2A receptors leads to the release of dopamine from dopaminergic neurons [87,88]. This event, a result of the indirect activation of dopaminergic pathways through the stimulation of serotonergic neurons and interaction with glutamatergic receptors, suggests the potential for significant changes in subjective perception and mood, adding an additional layer to psychedelic effects [89]. However, psilocybin’s ability to influence dopamine release could contribute to the variability in individual responses to these substances, as well as the diversity of experiences reported during and after treatment [90]. In this context, it is crucial to consider that changes in neurotransmitter release not only occur in specific brain areas but also affect the functional connectivity between different brain regions. Psilocybin, by modulating neuronal activity, may alter normal communication patterns between these brain regions, which could be reflected in changes in overall brain connectivity [91]. These alterations in the neuronal network may be responsible for the unique psychedelic experiences and heightened introspection associated with psilocybin ingestion [91,92].

In this regard, functional Magnetic Resonance Imaging (fMRI) studies have provided valuable information on how psilocybin remodels brain connectivity, identifying distinctive patterns of functional connectivity that suggest increased interaction between brain regions that are not normally so interconnected [93]. This phenomenon is associated with the so-called default mode network, a system of brain regions that collaborate when the mind is at rest and not engaged in specific external tasks [94]. Psilocybin has an impact on this network which is essential for cognition and introspection. fMRI has revealed that psilocybin induces changes in default network activity, generating a decrease in activity in some areas and an increase in others [93,94]. On one hand, the reduction in activity in certain regions such as the Posterior Cingulate Cortex (PCC) and the Medial Prefrontal Cortex (MPFC) during the experience is associated with a temporary disconnection of the ego and dissolution of boundaries between the self and the environment [84]. This phenomenon, known as “ego dissolution”, has been linked to heightened introspection, allowing for a deeper exploration of one’s mind and emotions [95]. On the other hand, increased activity in certain areas of the default network, such as the Insula and the Dorsolateral Prefrontal Cortex (DLPFC), may be related to the amplification of internal and emotional experiences. Self-awareness, understood as the ability to recognize and reflect on one’s mental state, is modulated [96]. In the context of MDD treatment, this could suggest interrupting negative thought patterns, encouraging exploration of underlying thoughts, and facilitating positive changes [84,95,96].

**Figure 3:**
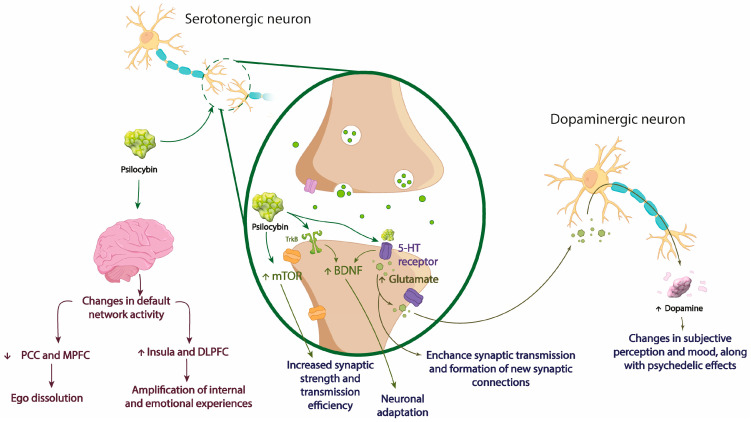
Representation of the mechanism of action of psilocybin. Psilocybin influences brain activity by modulating its networks activity, increasing some while decreasing others. Notably, it decreases activity in the PCC and MPFC, while elevating in the insula and DLPFC. Consequently, this process induces ego dissolution and intensifies internal and emotional experiences, respectively. It also binds to 5-HT receptors, leading to increased glutamate levels. This elevation in glutamate enhances synaptic transmission and promotes synaptic plasticity. Additionally, it also activates BDNF and mTOR second messengers’ pathways, inducing the neuronal adaptation and the incrementation of synaptic strength and transmission efficiency. Likewise, TrkB is activated, leading to an increase in BDNF release. Furthermore, heightened glutamate release stimulates dopamine release in dopaminergic neurons, eliciting mood changes and psychedelic effects. BDNF: brain-derived neurotrophic factor; DLPFC: dorsolateral prefrontal cortex; mTOR: mammalian target of rapamycin; MPFC: medial prefrontal cortex; PCC: posterior cingulate cortex; TrkB: tropomyosin receptor kinase B; 5-HT: serotonin.

Furthermore, the activation of second messenger pathways, such as the signaling cascade associated with BDNF release and modulation of the mTOR pathway, could explain the rapid antidepressant response induced by psilocybin [97]. Psilocybin, by activating 5-HT_2A_ receptors, promotes BDNF release, instigating molecular events that lead to the formation of new synapses by stimulating dendritic growth and branching, as well as facilitating the formation of synaptic spines [98]. Consequently, neuronal adaptation is promoted, catalyzing synaptic plasticity and its impact on the central nervous system’s (CNS’s) ability to adapt to changes in the environment. Additionally, mTOR activation stimulates protein synthesis, providing the biological basis for rapid and sustained changes in neuronal morphology and functionality (increased synaptic strength and transmission efficiency) that correlate with observed antidepressant effects [99]. Moreover, activation of the TrkB, necessary in neurotrophic signaling, is linked to BDNF release, inducing cellular responses that include neuronal survival and differentiation [100] (Figure 3).

Careful integration of the psychedelic experience into a therapeutic environment is crucial to maximize benefits and manage risks associated with the use of psychedelic substances like psilocybin in MDD treatment. This involves providing a supportive space before, during, and after treatment. Before the experience, preparation includes discussions about expectations, therapeutic goals, and psychological readiness. Subsequently, the integration phase involves working with the individual to reflect on the experience, process emotions, and apply them to everyday life [101]. It is essential to emphasize that integration focuses not only on the positive aspects of the experience but also addresses any challenges or discomfort that may arise.

##### Psilocybin and Mitochondria 

Mitochondria are also directly linked through the activation of 5-HT_2A_ receptors. Psilocybin has been shown to affect ATP levels in neocortical cells, indicating a direct impact on mitochondrial function [102]. This phenomenon is mediated by the regulator sirtuin 1 (SIRT1), which plays a crucial role in improving oxidative phosphorylation efficiency and mitochondrial respiratory capacity. Beyond mitochondrial function, psilocybin may influence mitochondrial biogenesis, a vital process involving the coordinated synthesis and assembly of new mitochondria [103]. The coactivator 1α of the peroxisome proliferator-activated receptor gamma (PGC-1α) and SIRT1 emerge as key players in regulating this process [102,103,104]. Additionally, activation of 5-HT_1A_ receptors, another component of the complex serotonergic system, has been observed to affect mitochondrial transport in hippocampal neurons [105]. While the specific connection between psilocybin and this phenomenon requires further detailed exploration, these findings highlight the multifaceted complexity of interactions between psychedelics and mitochondrial processes.

#### 3.1.4. Anti-Inflammatory Drugs and Cytokine Inhibitors 

The relationship between inflammatory processes and MDD has sparked growing interest. Numerous clinical studies have investigated the potential efficacy of nonsteroidal anti-inflammatory drugs (NSAIDs) and cytokine inhibitors in the treatment of MDD, revealing a connection between inflammation and depressive symptoms [106]. Although the underlying mechanism is not fully understood, various explanations have been proposed to comprehend the link between the anti-inflammatory response of NSAIDs and the improvement of MDD.

NSAIDs stand out for their ability to modulate the activity of cyclooxygenase enzymes (COX), particularly COX-1 and COX-2 [107]. These enzymes are essential in the cascade of prostaglandin synthesis, functioning as mediators in inflammatory responses. The selective inhibition of these enzymes by NSAIDs implies a reduction in prostaglandin formation, with significant implications in mitigating the inflammatory response. COX-1 is expressed constitutively in many tissues and is involved in normal physiological functions, such as gastric protection [108]. COX-2, on the other hand, is induced in response to inflammatory stimuli. The inhibition of COX-2 has been particularly relevant in the anti-inflammatory context. It has been suggested that, in addition to reducing inflammation, the inhibition of COX-2 could have direct effects on the CNS, affecting neurotransmitter release and modulating neuronal communication [109]. 

One of the key elements allowing for the direct impact of COX-2 inhibition on the CNS is the ability of certain NSAIDs to cross the blood-brain barrier (BBB). The BBB, which protects the brain by regulating the passage of substances from the blood, allows certain NSAIDs to reach glial cells and neurons in the CNS [110]. Additionally, it has been proposed that the inhibition of COX-2 could affect neurotransmitter release in brain synapses. Glial cells, particularly astrocytes, regulate neuronal homeostasis and neurotransmitter release. Therefore, COX-2 inhibition could modulate the activity of these astrocytes, influencing the release of neurotransmitters such as serotonin, dopamine, and glutamate [108,109,110]. The relationship between COX inhibition and depression is based on the interconnection between inflammation and mental health [111]. This relationship is not limited solely to physiological aspects but also encompasses emotional and cognitive dimensions. Various studies have suggested that COX-2 inhibition could directly interact with specific neurotransmitter systems [112]. For instance, serotonin could be particularly sensitive to alterations mediated by COX-2 inhibition. Consequently, the ability to modulate serotonin could have implications in mood regulation [113]. Additionally, elevated levels of prostaglandins have been associated with inflammatory processes, and chronic inflammation has been linked to the pathophysiology of depression. By blocking COX and reducing prostaglandin production, NSAIDs could exert a modulating effect on systemic inflammation and, consequently, depressive symptoms [112]. Preclinical studies have indicated that COX-2 inhibition may have neuroprotective effects by modulating neuronal excitability and reducing excitotoxicity [114]. Furthermore, it has been proposed that the regulation of prostaglandins in the brain can influence synaptic plasticity, a key component in MDD [114]. In addition to COX inhibition, NSAIDs have demonstrated significant effects on the regulation of proinflammatory cytokines. TNF-α and Interleukin-6 (IL-6), in particular, are important targets [115]. These cytokines are known for their involvement in the inflammatory cascade and have been identified as relevant biomarkers in the context of MDD, as elevated cytokine levels may trigger imbalances in neurotransmitter signaling, contributing to the manifestation of depressive symptoms [116]. Consequently, it has been suggested that these cytokines can impact the function of neurotransmitters such as serotonin and dopamine [117]. Therefore, by reducing cytokines, NSAIDs could positively influence neuronal homeostasis and communication, and thus modulate depressive symptoms. Various clinical trials that included patients with MDD and elevated levels of TNF-alpha demonstrated a significant decrease in depressive symptoms [118] with NSAID administration. This evidence supports the hypothesis that modulating proinflammatory cytokines with NSAIDs may have therapeutic implications for MDD [119].

**Figure 4:**
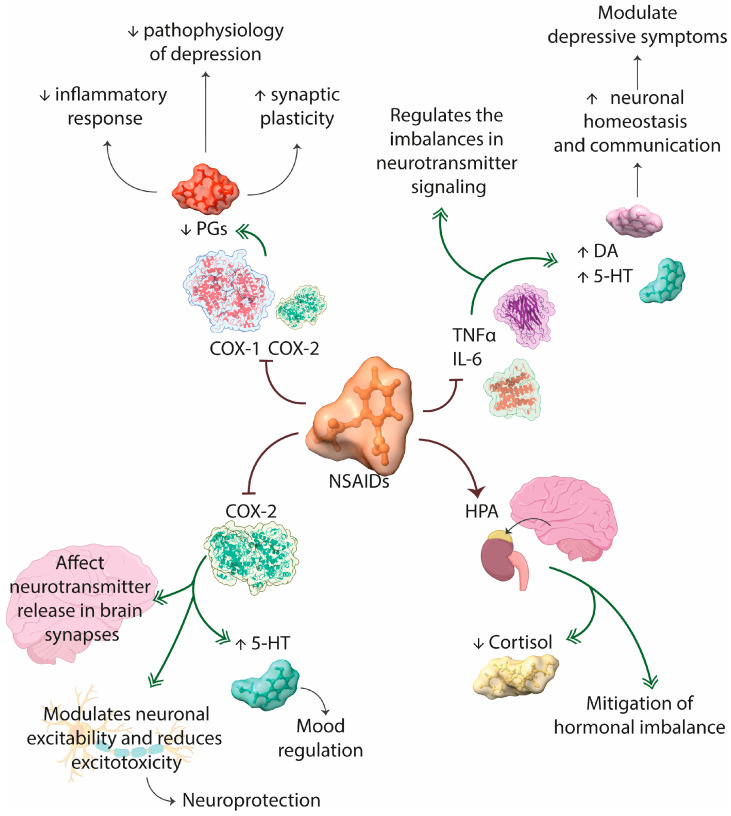
Schematic representation depicting the mechanism of action of NSAIDs. NSAIDs inhibit the enzyme COX, leading to decreased prostaglandin PGs levels, resulting in reduced inflammation, enhanced synaptic plasticity, and alleviating pathophysiology of depression. Moreover, inhibition of COX-2 by NSAIDs leads to specific effects such as increased 5-HT levels, aiding mood regulation, modulation of neurotransmitter release in brain synapses, excitability modulation, and reduced excitotoxicity, ultimately contributing to greater neuroprotection. Additionally, NSAIDs inhibit pro-inflammatory cytokines TNF and IL-6, aiding in regulating neurotransmission imbalances and increasing both dopamine and 5-HT levels, thus improving neuronal homeostasis by modulating depressive symptoms. Furthermore, NSAIDs also impact the hypothalamic-pituitary-adrenal axis, modulating certain hormones such as cortisol. COX: cyclooxygenase; IL-6: Interleukin-6; NSAIDs: nonsteroidal anti-inflammatory drugs; 5-HT: serotonin; TNF: tumor necrosis factor.

Moreover, chronic inflammation can have a significant impact on the hypothalamic-pituitary-adrenal (HPA) axis, a system involved in the physiological stress response. The HPA axis regulates the release of hormones, such as cortisol, which is found at elevated levels in individuals with MDD [120]. Thus, NSAIDs, by reducing the inflammatory burden, could contribute to normalizing the activity and sensitivity of the axis, mitigating the hormonal imbalances associated with depression (Figure 4).

The success observed in clinical trials underscores the relevance of future research exploring personalized therapies based on the specific modulation of proinflammatory cytokines. Understanding individual variations in the inflammatory response could lead to more precise and effective strategies for addressing MDD. Despite the potential benefits, it is essential to consider the possible side effects of cytokine modulation, as these molecules play vital roles in the immune response.

##### Anti-Inflammatory Drugs and Mitochondria

Anti-inflammatory drugs, such COX inhibitors and lipoxygenase inhibitors, have been used in clinical practice to modulate the inflammatory response, and it has been suggested that they may influence mitochondrial function [121]. However, the underlying molecular mechanisms of this interaction are not fully understood and continue to be the subject of intensive research. In terms of molecular mechanisms, it has been demonstrated that chronic inflammation induces the release of proinflammatory cytokines, such as TNF-α and interleukins, which can trigger the production of ROS in cells [115]. These ROS can have detrimental effects on mitochondrial function by damaging critical components of the mitochondrial machinery, such as electron transport chain proteins, thereby compromising the energy efficiency of mitochondria [122]. Anti-inflammatory agents, such as COX inhibitors, may counteract these effects by reducing ROS production and preserving the functional integrity of mitochondria, which potentially could mitigate the progression of mitochondrial dysfunction associated with chronic inflammation [121].

From a therapeutic perspective, understanding how anti-inflammatory compounds affect mitochondrial function offers new opportunities for the development of therapeutic strategies targeted at diseases where inflammation and mitochondrial dysfunction are contributing factors. Specifically, it has been observed that aspirin, in addition to its well-known anti-inflammatory properties, may have a direct impact on mitochondrial function by activating AMP-activated protein kinase (AMPK) cells [123]. AMPK is a master kinase protein that regulates cellular energy homeostasis and plays a fundamental role in mitochondrial biogenesis and function. Activation of AMPK by aspirin may result in improved mitochondrial function and increased cellular energy efficiency, which could have significant implications for the treatment of diseases associated with inflammation and mitochondrial dysfunction [123].

Therefore, the identification of anti-inflammatory compounds that can positively modulate mitochondrial function represents a promising area of research that could have significant clinical implications in the future.

### 3.2. Non-Pharmacological Treatment

#### 3.2.1. Transcraneal Magnetic Stimulation 

In the treatment of major depression, Transcranial Magnetic Stimulation (TMS) has emerged as a cutting-edge technique, harnessing the ability to modulate neuronal activity through non-invasive magnetic fields [124]. This revolutionary approach is grounded in profound neurophysiological principles, where neuronal depolarization, neurotransmitter release, and activation of intracellular pathways configure an intricate network of events. TMS is a non-invasive technique that relies on the modulation of neuronal activity through magnetic fields. The application of these magnetic fields induces electric currents in the brain based on Faraday’s law [125]. The parameters are adjusted during the sessions and may vary depending on the protocol. The technique is performed by placing an electromagnetic coil on the patient’s scalp. The choice of the coil depends on various factors such as the depth of stimulation, specificity of the area, and patient comfort, with the circular coil and figure-eight coil being the most used [126].

- Circular Coil: A round-shaped coil that allows for the induction of a homogeneous magnetic field with a preference for superficial cortical areas. This type of coil is often selected for more superficial or generalized stimulations in a broader brain region [126].
- Figure-Eight Coil: It has two loops forming a figure-eight structure, enabling greater focus of stimulation in the area where the two loops cross. This type of coil is usually chosen when aiming to direct magnetic pulses to specific brain regions, minimizing undesired stimulation in surrounding areas [126].

The placement of the coil is crucial for effectively inducing magnetic pulses to specific brain areas. Methods used are selected based on resource availability, required precision, patient anatomy, and the nature of the treatment, with precise placement being essential for the efficacy and safety of transcranial magnetic stimulation.

- 10–20 EEG System Method: Utilizes the International 10–20 EEG system to identify specific areas of the skull. Distances between electrodes on the scalp are measured to determine the coil’s location. Its use is common for routine clinical applications [127].
- Neuronavigation: This method employs medical images such as magnetic resonance imaging (MRI) or computed tomography (CT) to create a three-dimensional map of the brain. This real-time map guides the exact placement of the coil, allowing for precise positioning based on each individual’s brain anatomy. Its use is common for advanced clinical applications [128].
- Anatomical Methods: Points such as the central sulcus are used to orient the coil, with selected points depending on the stimulation goal. Stereotactic coordinates are employed to specify the three-dimensional position of the coil in relation to brain anatomy [126].

The stimulation region plays a critical role in the efficacy and specificity of the treatment. The DLPFC is one of the most studied and utilized regions in transcranial electrical stimulation for the treatment of major depression [129]. The DLPFC, located in the frontal part of the brain (lateral and superior surface), is involved in decision-making, essential cognitive functions, executive functions, and emotions. In individuals with major depression, neurophysiological and neuroanatomical changes have been observed in this region, revealing neuronal hypoactivity and patterns of functional disconnection. Functional neuroimaging studies have documented a decrease in metabolic activity in the DLPFC, associated with difficulties in cognitive tasks and compromised emotional regulation [129]. Additionally, functional disconnection suggests alterations in neuronal coordination. In terms of structural changes, reductions in the cerebral volume of the DLPFC have been identified, linked to possible neuronal loss or morphological alterations [128,129]. Alterations in receptor density, particularly of neurotransmitters such as serotonin and dopamine, have also been observed, influencing neurotransmission and contributing to mood changes [130]. As a result of the relationship between the DLPFC and depression, along with its inherent neuronal plasticity, this region emerges as a potential therapeutic target. However, the study of various brain areas has also been addressed to better understand the complexity of the neurobiology of this disorder and explore therapeutic potential in other brain regions (Table 1).

On one hand, the starting point of TMS action lies in neuronal depolarization. This phenomenon induces temporary changes in the membrane potential of neurons, triggering the opening of ion channels and the release of neurotransmitters at nearby synapses [137]. This event generates a cascade of intracellular events that contribute to the complex network of neurochemical interactions. A notable increase in glutamate release, the primary excitatory neurotransmitter in the brain, stands out as a focal point [138]. This increase not only drives neuronal activity but also establishes connections with monoaminergic neurotransmitters such as serotonin, norepinephrine, and dopamine, all crucial in mood regulation and the pathophysiology of depression. The complexity of these neurochemical interactions lies in the ability of TMS to selectively modulate different neuronal populations and neurotransmitter systems [139]. On the other hand, TMS also activates intracellular pathways that trigger molecular-level responses. Among these pathways, there is an increased release of BDNF, an essential neurotrophic protein in survival and synaptic plasticity, suggesting a profound influence on neuronal adaptation [140]. At the molecular level, BDNF signaling involves the activation of specific receptors such as TrkB. TrkB activation triggers intracellular signaling cascades, including the phosphatidylinositol 3-kinase (PI3K)/protein kinase B (Akt) pathway and the Mitogen-Activated Protein Kinase/Extracellular Signal-Regulated Kinase (MAPK/ERK) pathway [141]. These pathways are involved in the regulation of gene expression, protein synthesis, and cell survival, mediating the long-term effects of TMS on neuronal adaptation. Another key component in TMS-induced intracellular signaling is the activation of protein kinases. Protein kinases, such as MAPK and Protein Kinase C (PKC), participate in the transduction of intracellular signals and are associated with the modulation of synaptic plasticity in the neuronal response to external stimuli. These protein kinases play a crucial role in long-term neuronal adaptation, connecting magnetic stimulation with profound molecular changes [142]. Furthermore, the influence of TMS on synaptic plasticity is not limited to the immediate synapse affected by stimulation. It extends to distant neuronal connections, triggering molecular adaptations that redefine brain connectivity [143]. At the molecular level, extended synaptic plasticity manifests in changes in gene expression, protein synthesis, and modification of synaptic receptors. TMS, by altering synaptic efficacy, induces intracellular events that have implications for long-term synaptic structure and function. This process contributes to sustained neuronal adaptation, essential for the observed therapeutic effects in the treatment of MDD [143,144].

In conclusion, TMS not only represents a technical advancement in neuropsychiatric intervention but also opens new perspectives in our understanding of the neurobiology of MDD [124,138,143]. From initial depolarization to intracellular modulation and extended synaptic plasticity, each component of this process reflects the intrinsic complexity of the brain’s response to magnetic stimulation. The specificity in coil placement, supported by advanced methods such as neuronavigation, underscores the importance of anatomical precision in therapeutic efficacy. As we progress in the convergence of clinical research and molecular neuroscience, TMS stands as a promising pillar in the treatment of major depression, igniting the spark of innovation at the intersection of mind and technology.

#### 3.2.2. Deep Brain Stimulation

Deep Brain Stimulation (DBS) is an advanced neurosurgical technique involving the insertion of electrodes into specific areas of the brain to modulate neuronal activity through precise application of electrical current [145]. This approach is primarily employed in patients with TRD, where conventional treatments such as pharmacotherapy and psychotherapy have been ineffective [146].

The DBS procedure for depression follows a meticulous protocol, beginning with comprehensive patient evaluations, including psychiatric and neuropsychological assessments, as well as brain imaging to identify target regions. Implantation surgery is conducted under general anesthesia with real time imaging assistance, such as magnetic resonance imaging or computed tomography, to ensure accurate electrode placement [147]. Once electrodes are inserted, stimulation programming is initiated. This stage involves adjusting key parameters gradually to achieve optimal therapeutic effects and minimize side effects. Patients are closely monitored post-surgery to assess DBS efficacy and make programming adjustments as necessary to maintain optimal outcomes.

The selection of the target brain region is crucial and may include different areas related to depression.

- Subcallosal Cingulate Cortex (SCC): Associated with the regulation of negative mood states, with increased activity observed. It is interconnected with regions involved in emotion processing and motivation, with reciprocal pathways to other subcortical regions. The goal of DBS is to reduce hyperactivity and interrupt negative mood states. Studies have shown that remission rates are initially reduced but significantly increase in the long term [148].
- Lateral Habenula (LHb): Implicated in negative mood states and found to be activated with reduced volume in patients with MDD [148].
- Ventral Capsule and Ventral Striatum (VC and VS): Linked to mood regulation and reward. Due to varied outcomes in studies, a more extensive optimization phase is suggested.
- Medial Forebrain Bundle (MFB): Associated with the reward circuitry. Various studies have shown that its modulation can produce significant changes in anhedonia both in the short and long term [149].

In the context of depression, DBS is believed to exert its therapeutic effect by modulating neuronal activity in specific brain regions known to be involved in mood and emotion regulation [150]. Though the exact mechanism is not fully understood, it is thought to act through various mechanisms, including stimulation of reward and limbic circuits, inhibition of excessive activity in hyperactive brain regions, and increased neurotransmitter release such as serotonin, dopamine, and norepinephrine [145,147]. Additionally, DBS has been suggested to induce long-term changes in brain structure and function, promoting neuroplasticity and recovery of normal neuronal function [151]. Therefore, DBS represents a promising therapeutic option for patients with treatment-resistant depression. However, further research is needed to overcome current limitations, better understand the mechanisms of action, and optimize treatment efficacy and safety. With a multidisciplinary approach integrating neuroscience, psychiatry, and biomedical engineering, it is possible to improve patient quality of life and advance the field of neurostimulation for psychiatric disorders.

##### Non-Pharmacological Treatment Stimulation and Mitochondria

Although the exact mechanism by which TMS and DBS exerts its therapeutic effects is not fully understood, there is evidence suggesting that it may impact mitochondrial function. One way in which TMS and DBS is thought to affect mitochondrial function is by promoting neuroplasticity [140,151]. TMS has been associated with increased neuroplasticity by stimulating the release of neurotransmitters such as glutamate and BDNF [152]. It has been demonstrated that BDNF is involved in regulating mitochondrial function by increasing the expression of genes related to mitochondrial biogenesis and function, as well as by enhancing the activity of key mitochondrial enzymes such as cytochrome c oxidase [153]. Additionally, BDNF has been suggested to protect mitochondria against oxidative stress by activating signaling pathways that regulate calcium homeostasis and antioxidant response [154]. It has also been proposed that TMS and DBS modulates cerebral metabolic activity and consequently mitochondrial function. Preliminary studies have shown that TMS and DBS can increase metabolic activity in specific brain regions such as the DLPFC and the anterior cingulate cortex (ACC) [135,150]. This modulation of cerebral metabolic activity could impact mitochondrial function by increasing the energy demand of neurons in these regions [151]. Consequently, an increase in energy demand could induce greater ATP synthesis and ultimately lead to an improvement in mitochondrial function. Additionally, it has been suggested that TMS may influence the activity of key metabolic enzymes that regulate glycolysis and lipid metabolism, processes closely related to mitochondrial function [155]. Furthermore, DBS could modulate important cellular signaling pathways for neuronal survival and function, such as the IGF pathway [156] and the protein kinase B (Akt) pathway. These signaling pathways can influence gene expression and enzyme activity related to mitochondrial function [157]. For example, activation of the IGF pathway has been shown to increase mitochondrial biogenesis and improve mitochondrial function by upregulating the expression of mitochondrial genes and the activity of enzymes involved in cellular respiration and ATP production [82]. Additionally, Akt has been suggested to protect mitochondria against oxidative stress by regulating the activity of anti-apoptotic and antioxidant proteins in the outer mitochondrial membrane [158].

In summary, although further research is still needed to fully understand how TMS and DBS affect mitochondrial function, existing evidence suggests that these techniques may promote neuroplasticity, modulate cerebral metabolic activity, and regulate important cellular signaling pathways, all of which may have beneficial effects on mitochondrial function and, ultimately, on neuronal function and individual well-being.

## 4. Future Perspectives

Despite sustained efforts to develop pharmacological and non-pharmacological treatments for MDD, there is still a need for deeper understanding of its underlying neurobiology. This understanding will enable the application of more precise and effective treatments, leading to significant improvements in clinical outcomes and patients’ quality of life (Table 2).

Early and accurate detection of MDD is crucial for effectively intervening in its clinical course. The integration of clinical, neurobiological, and biochemical data through the identification of biomarkers has been established as a key tool in this area [5]. These biomarkers provide valuable insights into the underlying pathophysiological processes and can be used to predict responses to different treatment modalities more accurately [42,45,159]. However, advances in the diagnosis and treatment of MDD face considerable challenges, including the need to investigate the long-term effects of emerging pharmacological therapies [78,144]. Although conventional antidepressants remain the cornerstone of treatment, there is a growing interest in the development of alternative therapies that address the limitations of current approaches. These new treatments seek not only to alleviate symptoms but also to address the neurobiological alterations associated with MDD [5]. In this context, various promising pharmacological options are being explored. Psilocybin has shown promising results in preliminary studies as a treatment for TRD [84,88,91]. Its ability to affect serotonin receptors in the brain can induce altered states of consciousness that promote introspection and emotional well-being [87]. Additionally, anti-inflammatory agents are being investigated due to the close association between inflammation and depression in some cases [112]. These drugs aim to reduce the body’s inflammatory response, which is believed to contribute to the development and maintenance of depression in some patients. [111] Another promising and approved option is ketamine and its derivative esketamine. These options have demonstrated a rapid and significant antidepressant effect in clinical studies, even in patients who do not respond to other treatments [46,71,77,78,85,160]. Their unique mechanism of action involves rapid modulation and facilitation of synaptogenesis in the brain [53,57,73,161]. However, further research is needed to fully understand their long-term efficacy, identify patients who would benefit most, and minimize potential side effects.

Non-pharmacological therapies, such as TMS and DBS, have garnered significant interest. This technique offers the ability to selectively modulate neuronal activity in specific brain regions, which may influence the circuits affected in MDD [140,143,147]. Despite these techniques being considered for patients with treatment-resistant depression, there remains a need to identify more precise selection criteria to predict treatment responses and optimize outcomes [145]. Additionally, while several mechanisms of action have been proposed, its functioning at the molecular and cellular levels is still not fully understood, necessitating further studies to elucidate these mechanisms and improve treatment efficacy. Moreover, programming stimulation is a complex process that requires additional research to develop more sophisticated and personalized algorithms, ensuring optimal therapeutic outcomes and minimizing side effects [162].

Furthermore, progress is being made in understanding the interaction between pharmacological and non-pharmacological treatments, suggesting that a combined therapeutic strategy could offer synergistic benefits. This combination of therapeutic approaches aims to address multiple neurobiological pathways involved in MDD, which may improve treatment response and reduce disease burden. There is great potential for understanding the mechanisms underlying TRD through research on mitochondria [22]. These cellular organelles serve not only as possible sources of useful biomarkers, but also as potential prospective treatment targets. An increasing amount of research indicates that mitochondrial dysfunction may be a key factor in the pathogenesis of MDD [39,105]. Thus, finding biomarkers linked to mitochondrial activity may transform the early detection and prognosis of MDD that is resistant to treatment, eventually opening the door for the creation of more specialized and efficient therapeutic approaches [163].

In summary, ongoing research into the neurobiology and treatment of MDD has the potential to radically transform clinical care and improve outcomes for patients. However, further studies are needed to translate these advances into effective and personalized therapeutic strategies that address the complex interactions between the neurobiological systems implicated in MDD.

## Figures and Tables

**Table 1:** Different brain regions and their function in MDD.

Area	Localization	Function	
Ventromedial Prefrontal Cortex	Subcortical region	Involved in the evaluation of rewards and punishments	[131]
Anterior Cingulate Gyrus	Frontal lobe	Involved in the regulation of emotional and stress responses	[132]
Dorsolateral Parietal Lobe	Parietal lobe	Involved in attention and working memory	[133]
Posterior Cingulate Cortex	Posterior region	Involved in the regulation of pain and emotion responses	[134]
Nucleus Accumbens	Subcortical region	Involved in the reward system, related to anhedonia	[135]
Ventral Tegmental Area	Brainstem	Associated with dopamine release; involved in the regulation of motivation and pleasure	[136]

**Table 2:** SWOT analysis for the study, treatment, and management of MDD.

Mayor Depressive Disorder	
Strengths	Opportunities
Detectable peripheral biomarkers help in diagnosis and prognosis. Therapeutic diversity options. Rapid action of new therapeutic targets.	Advancements in research of therapeutic targets and diagnosis biomarkers with special focus on mitochondrial respiration Development of new therapies. Treatment personalization. Increased awareness and destigmatization of the disease.
Weaknesses	Threats
Clinical and pre-clinical research in specific field is required. Lack of understanding of mechanisms of action of new therapeutic targets. Safety and tolerability of new drugs. Cost and accessibility.	Resistance to change from healthcare professionals and patients. Regulation and approval of new treatments. Uncertainty about long-term efficacy and safety.
